# Incidental findings on 18-FDG PET–CT in head and neck cancer. A retrospective case-control study of incidental findings on 18-FDG PET–CT in patients with head and neck cancer

**DOI:** 10.1007/s00405-018-5203-1

**Published:** 2018-12-10

**Authors:** Elizabeth Casselden, Fintan Sheerin, Stuart C. Winter

**Affiliations:** 1 Department of Ear, Nose, and Throat Surgery, Oxford University Hospital NHS Foundation Trust, Oxford, UK; 2 Department of Neuroradiology, Oxford University Hospital NHS Foundation Trust, Oxford, UK; 3 Nuffield Department of Surgery, University of Oxford, Oxford, UK

**Keywords:** Positron emission tomography computed tomography, Head and neck neoplasms, Fluorodeoxyglucose F18, Incidental findings, Metastases

## Abstract

**Purpose:**

Use of 18-FDG PET–CT is increasing in patients with head and neck cancer, enabling the identification of metastases or synchronous malignancies, but also ‘incidental’ disease. We aimed to establish the rate of ‘incidental’ findings resulting from 18-FDG PET-specific imaging, that would not have been otherwise identified on other imaging, in patients with head and neck cancer undergoing staging or surveillance of disease.

**Methods:**

18-FDG PET–CT was performed for investigation or surveillance. Case notes were reviewed retrospectively. Unexpected findings identifiable on CT imaging alone, or by FDG-PET were recorded. For those only identifiable with FDG-PET, findings were divided into either ‘incidental’ or ‘intentional’, and benign or malignant.

**Results:**

93 patients underwent 18- FDG PET–CT. 86.0% had new pathology identified. 3.2% had a new malignancy identified. 37.6% had new findings on FDG-PET that would not have been identified on CT alone: 5.4% had ‘intentional findings’ (metastasis), and 32.3% had ‘incidental findings’ (synchronous malignancy or benign). 1.1% had a new malignancy on FDG-PET alone.

**Conclusions:**

Intentional and incidental findings are likely on 18-FDG PET–CT. Whilst important for patient management, there is an associated emotional and financial cost, which needs acknowledgement and further investigation.

## Introduction

Annually, head and neck cancer (HNC) affects 700,000 people worldwide and over 9000 in the UK [1]. Within the UK, tumours of the oropharynx (tonsil and tongue base) have seen a twofold increase in incidence over the last 20 years largely attributed to human papillomavirus (HPV) [2]. Also during this time there has been a 30% increase in oral cancer with increases predicted to continue [3].

The role of 18-fluorodeoxyglucose positron emission tomography–computed tomography (18-FDG PET–CT) has continued to increase, in part because the sensitivity and specificity for identifying the primary site and extent of nodal disease is improved compared to other forms of radiological investigation. This is due to the combination of structural assessment using the CT component, alongside the metabolic component using the FDG tracer.

This is of particular importance when the primary site is not identified at the initial investigation. Indeed, NICE advocates 18-FDG PET–CT early in the diagnostic pathway for these patients [4]. Also in those patients treated with primary CRT, the recent study by Mehanna et al. has shown that the results of 18-FDG PET–CT can be used to guide the management of the neck and avoid a neck dissection in many situations [5]. This is advantageous both in terms of morbidity to the patient, and cost to the health service.

A further advantage of 18-FDG PET–CT is that the whole body is imaged. This is in contrast to traditional imaging techniques which typically cover only the primary site and chest, with or without the upper abdomen. In particular, chest imaging is typically performed looking for either a synchronous lung cancer or metastatic spread, both of which would influence management options.

It is also recognised that 18-FDG PET–CT may identify incidental findings both within the neck and chest, but also out of the field of traditional imaging. Some of these findings will represent previously unrecognised malignant processes. Conversely many of these findings will represent *benign* ‘incidental’ findings.

These ‘incidental’ findings will often require additional investigations incurring an additional health economic burden as well as a psychological concern for the patient.

This is the first study in the UK HNC population to investigate the rate of incidental findings in patients undergoing 18-FDG PET–CT investigation. We aimed to ascertain the rate and nature of incidental findings reported on 18-FDG PET–CT in our population.

## Materials and methods

### Setting

Tertiary head and neck cancer centre in a UK teaching hospital.

### Study design

Inclusion criteria: This was a retrospective case-control study of consecutive patients presenting to the HNC unit over a 15-month period. Those that were investigated with 18-FDG PET–CT either as part of the investigation prior to definitive treatment or for monitoring of disease response were included in the study. Patients were excluded if they did not have a (suspected) malignant process of the head and neck, or had a thyroid malignancy.

A case file review was performed and information collected regarding age, sex, primary site, nodal disease, pathology final staging and, for oropharyngeal SCC, P16 status.

The 18-FDG PET–CT reports were analysed and findings, as interpreted by the reporting radiologist, recorded. Findings were reviewed by a further consultant radiologist as to whether they would have been found on CT (which would have been performed as part of staging in HNC patients), or if they were noted due to their FDG avidity.

New findings specifically found by the FDG-PET element of the scan, were divided into ‘intentional findings’ (i.e. distant metastases - excluding cervical lymphadenopathy), and ‘incidental findings’ (including incidental synchronous malignancies and other, presumed benign findings).

Specific recommendations for follow-up or further investigations of incidental findings as suggested by the reporting radiologist were recorded. Statistics were descriptive.

## Results

### Patient numbers and demographics

236 consecutive patient notes were reviewed, who presented to the HNC clinic as above. 119 had undergone 18-FDG PET–CT and were, therefore, included in the study. 26 further patients were excluded as above, leaving 93 patients in the study.

Of those included having 18-FDG PET–CT, the mean age was 60 (34–85). 75 (80.6% of patients undergoing 18-FDG PET–CT as above) were male. Table 1 shows the locations of tumours in the cohort.

**Table 1:** Site of primary tumour amongst the 93 patients included in the study

Tumour area	Number	Percentage
Oropharynx	56	60.2
Hypopharynx	9	9.6
Larynx	8	8.5
Sinonasal	4	4.3
Salivary gland	8	8.5
Cancer of unknown primary	5	5.3
Other	1	1.1
Lymphoma	5	5.3
Total	96	

Note, 3 patients had tumours involving multiple subsites

56 (60.2%) were diagnosed with an SCC of the oropharynx. Of these, 42 (75.0%) were positive for p16 and 5 were negative. There were nine patients for whom the p16 status could not be found. After investigation, five patients were classified as having cancer of the unknown primary (CUP). All were SCC in one or more neck nodes. Of the eight (8.5%) patients with salivary gland tumours, seven were in the parotid and one was in the submandibular gland. The histological diagnoses for these tumours were both benign and malignant: pleomorphic adenoma (2), Warthin’s tumour, SCC (2), neuroendocrine carcinoma, ductal carcinoma, basal cell adenocarcinoma. There was one (1.1%) malignant melanoma presenting in the ear.

The 93 patients underwent a total of 145 scans (mean 1.56/patient).

### Findings

**Fig. 1:**
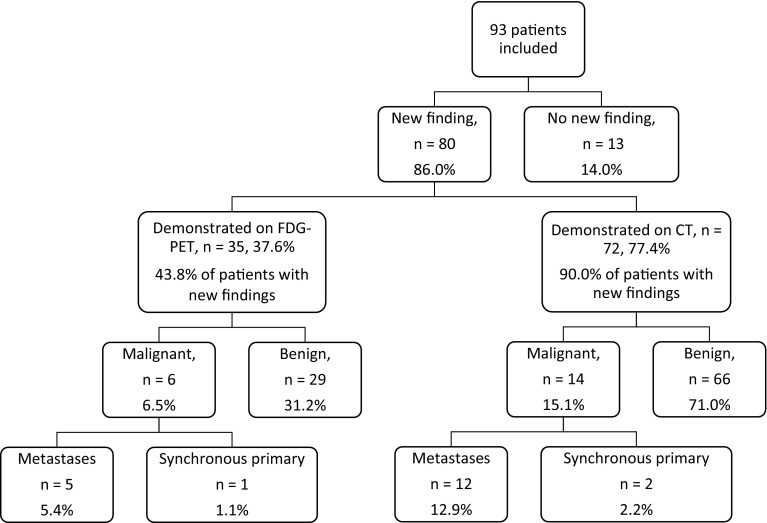
Summary of findings. Note percentages given are of total patients scanned (93) unless stated otherwise. Some patients had multiple findings

Of the 93 patients, 80 (86.0%) had a new finding on at least one scan that was beyond local associated lymphadenopathy. Figure 1 demonstrates how these have been classified. A total of three malignancies were identified (3.2%): oesophageal, thyroid and lung.

#### FDG-PET specific findings

72 patients (90.0% of those with new findings, 77.4% of those scanned) had findings that would have been found on CT imaging alone. 35 (43.8% of those with new findings, 37.6% of patients scanned) had at least one previously unknown finding that was specifically shown by FDG-PET. In the case of malignant findings, this included findings that would have been considered benign on CT imaging alone.

5 patients (5.4% of scanned patients, 6.3% of those with new findings, 14.3% of those with FDG-PET findings) had metastatic, i.e. ‘intentional findings’. These included bone, lung, and axillary and parotid nodes.

30 patients (32.3% of scanned patients, 37.5% of those with new findings, 85.7% of those with FDG-PET findings) had ‘incidental findings’. 29 of these patients had benign findings, which included colorectal changes (13, including polyps/low grade adenomas, a gastrointestinal stromal tumour, and inflammatory changes including diverticulitis and crohn’s disease); lung nodes, nodules and inflammatory/infectious changes (6); prostate changes (4); biliary inflammation (3); sebaceous cysts (2); reactive axillary nodes (2); arthropathy (2); contralateral tonsillar changes (1, proven to be benign on biopsy); oesophagitis (1); thyroiditis (1). In one patient (1.1% of all patients scanned), FDG-PET demonstrated a hot thyroid nodule that was not seen on CT and was subsequently found to be a Thy 5 papillary cancer.

#### Findings requiring further investigation

To better determine ‘incidental findings’, in some cases the reporting radiologist was noted to have made recommendations as to further investigations. In those with FDG-PET identified ‘incidental findings’, these included biopsies/fine needle aspiration (3), colonoscopy/flexible sigmoidoscopy (9), PSA (3), and CT chest (3), MRI brain (1), making a total of 19 patients (20.4% of all patients scanned).

#### CT identified findings

Two further patients had a previously undiagnosed synchronous malignancy identified—these were oesophageal and lung. Of note, the oesophageal lesion was identified as malignant on CT imaging, but the FDG-PET avidity heightened suspicion in this patient.

Twelve (12.9%) patients had metastatic disease that would have been identified on CT imaging alone. Sites included bone, adrenal, lung, liver, parotid, axilla, contralateral tonsil and other lymphoma sites.

Benign findings that would have been identified on CT imaging alone included: lung nodes/nodules and other changes including lung granulomas, emphysema, atelectasis, pneumonia, pleural plaques; hepatobiliary and pancreatic disease; diverticular disease; coronary calcification; hiatus hernia; abdominal wall hernias; orthopaedic changes; renal cysts and urolithiasis; adnexal/ovarian cysts and uterine fibroids.

## Discussion

### Key findings

HNC is increasing in incidence with patients presenting at earlier ages. Assessment of patients increasingly involves 18-FDG PET–CT. Advantages include improved sensitivity and specificity compared to other imaging modalities [6]. 18-FDG PET–CT has also been shown to guide the management of the neck following chemoradiotherapy [6]. 18-FDG PET–CT also has the advantage of whole body assessment, with both structural and metabolic information. This added information also has a recognised incidence of additional findings, which may include metastases, synchronous primary tumours and benign incidental findings.

86.0% of patients had a new finding, of which 43.8% were specifically found on FDG-PET images. An increased cost to the health service is raised, as 20.4% were recommended to undergo further investigation. Only one (1.1% of all patients) was found to be malignant following further investigation. Incidental findings, or the possibility of incidental findings, may raise health anxiety in patients, particularly as they are already likely to be anxious whilst undergoing investigation into HNC. Our results show that the majority of incidental findings specific to FDG-PET are benign, which may be of reassurance to patients. Clearly any counselling would need to be specific to the nature of findings in question.

The FDG-PET element of the scan was noted to add confidence to a malignant/metastatic new finding on CT in some cases. For example, differentiating benign pulmonary nodules from metastasis, or aiding in the identification of an oesophageal tumour, which although commonly demonstrated by CT, was considerably more conspicuous with FDG-PET.

### Comparisons with other studies

This is the first UK study to report on incidental findings on 18-FDG PET–CT in HNC patients. The authors were able to find one other study investigating the same topic specifically in head and neck cancers. This was undertaken in the USA, where Britt et al. report rates of 14.6% suspected malignancy, with 4.1% (12/293) confirmed new unrelated malignancy (5 were thyroid cancer; 2, lung cancer; 2, gastrointestinal; and 1 each of HNC, lymphoma, and genitourinary cancer) [7], a rate which is similar to our UK cohort.

In a large study in China, Wang et al. report a rate of 8% proven, unrelated/unexpected second malignancies in patients undergoing 18-FDG PET–CT who underwent ‘complete’ follow-up [8]. The study regards 18-FDG PET–CT performed for various malignancies, not just confined to the head and neck. In the USA, Beatty et al. report a rate of 11.5% (3/26 patients reported) with incidental synchronous primary malignancies in patients with head and neck primaries, and an overall rate of incidental synchronous primary cancers in all investigated primaries of 15% [9]. These rates are somewhat higher than those presented in our study, even when only considering head and neck malignancies.

### Implications for research

With the increasing use of 18-FDG PET–CT this study represents important information when planning treatments. The reference cost of the imaging does not account for the additional procedures needed to investigate the incidental findings. In a small percentage these findings are malignant processes that require management. However, the majority of findings are benign processes. It is possible that in certain situations these findings could result in delayed cancer treatment whilst those investigations are performed, and this should be investigated in further, larger studies. Furthermore, the psychological burden on the patient in instances such as this has not been measured but needs to be considered in future studies.

### Clinical applicability

The results presented in this UK-based study may facilitate the counselling of patients in clinic prior to undergoing 18-FDG PET–CT for HNC. With further investigation, the results may be applicable when planning provision and funding of healthcare resources.

## Conclusions

We have demonstrated an overall rate of synchronous malignant incidental findings on 18-FDG PET–CT performed for HNC that is similar to other authors, of 3.2%. We have demonstrated a rate of FDG-PET pickup of malignant findings that would not have been identified on CT imaging alone, of 1.1%.

In 43.8% of patients with new findings, FDG-PET identified disease that would not have been identified on CT imaging alone. Non-CT identifiable benign disease was found in 31.2%, and synchronous malignancy in 1.1%. Some benign findings underwent further investigation to confirm their benign nature. This raises a psychological and financial cost.

Whilst 18-FDG PET–CT is clearly of use in investigating proven or suspected HNC, due to its whole-body nature, other benign pathologies are likely to be found. This carries a psychological and financial burden to the patient and health service, which requires further investigation and should be borne in mind when counselling patients.
